# Information‐anchored sensitivity analysis: theory and application

**DOI:** 10.1111/rssa.12423

**Published:** 2018-11-16

**Authors:** Suzie Cro, James R. Carpenter, Michael G. Kenward

**Affiliations:** ^1^ University College London London School of Hygiene and Tropical Medicine, and Imperial College London UK; ^2^ University College London London School of Hygiene and Tropical Medicine UK; ^3^ Ashkirk UK

**Keywords:** Controlled multiple imputation, Deviations, Missing data, Randomized controlled trial, Sensitivity analysis

## Abstract

Analysis of longitudinal randomized clinical trials is frequently complicated because patients deviate from the protocol. Where such deviations are relevant for the estimand, we are typically required to make an untestable assumption about post‐deviation behaviour to perform our primary analysis and to estimate the treatment effect. In such settings, it is now widely recognized that we should follow this with sensitivity analyses to explore the robustness of our inferences to alternative assumptions about post‐deviation behaviour. Although there has been much work on how to conduct such sensitivity analyses, little attention has been given to the appropriate loss of information due to missing data within sensitivity analysis. We argue that more attention needs to be given to this issue, showing that it is quite possible for sensitivity analysis to decrease and increase the information about the treatment effect. To address this critical issue, we introduce the concept of *information‐anchored* sensitivity analysis. By this we mean sensitivity analyses in which the proportion of information about the treatment estimate lost because of missing data is the same as the proportion of information about the treatment estimate lost because of missing data in the primary analysis. We argue that this forms a transparent, practical starting point for interpretation of sensitivity analysis. We then derive results showing that, for longitudinal continuous data, a broad class of controlled and reference‐based sensitivity analyses performed by multiple imputation are information anchored. We illustrate the theory with simulations and an analysis of a peer review trial and then discuss our work in the context of other recent work in this area. Our results give a theoretical basis for the use of controlled multiple‐imputation procedures for sensitivity analysis.

## 1 Introduction

The statistical analysis of longitudinal randomized clinical trials is frequently complicated because patients deviate from the trial protocol. Such deviations are increasingly referred to as intercurrent events. For example, patients might withdraw from trial treatment, switch treatment, receive additional rescue therapy or simply become lost to follow‐up. Post deviation, such patients’ data (if available) will often no longer be directly relevant for the primary estimand. Consequently, such post‐deviation data are often set as missing; any observed post‐deviation data can then inform the missing data assumptions. Nevertheless, however the analysis is approached, unverifiable assumptions about aspects of the statistical distribution of the post‐deviation data must be made.

Recognizing this, recent regulatory guidelines from the European Medicines Agency Committee for Medicinal Products for Human Use (2010) and a US Food and Drug Administration mandated panel report by the National Research Council (2010) emphasize the importance of conducting sensitivity analyses. Further, the recent publication of the International Conference on Harmonisation of Technical Requirements for Registration of Pharmaceuticals for Human Use (2017) E9 (R1) addendum on estimands and sensitivity analysis in clinical trials raises important issues about how such sensitivity analyses should be approached. It highlights how in any trial setting it is important first to define the estimand of interest. This will inform what data are missing and how such missing data should be handled in the primary analysis. Sensitivity analysis, which targets the same estimand, should subsequently be undertaken to address the robustness of inferences to the underlying assumptions, including those made for the missing data.

We propose splitting sensitivity analyses for missing data into two broad classes. In both classes, one or more alternative sets of assumptions (or scenarios) are postulated and the sensitivity of the conclusions to these alternative scenarios is to be assessed. In our first class, the primary analysis model is retained in the sensitivity analysis. This enables the exclusive assessment of the effect of alternative missing data assumptions on the primary outcome of interest. For example, for our sensitivity analysis we may impute missing data under an assumption of data missing not at random and fit the primary analysis model to these imputed data. When performed by multiple imputation, class 1 sensitivity analyses are therefore uncongenial, in the sense described by Meng (1994) and Xie and Meng (2017), i.e. in brief, the imputation model and the analysis model are not the same, or conditionals of a single joint model. Conversely, in the second class, for each set of sensitivity assumptions an appropriate analysis model is identified and fitted. Hence, each such analysis model is consistent with its assumptions, which is why the analysis models generally change as we move from scenario to scenario.

In the first class of sensitivity analyses, the assumptions of the primary analysis model may be inconsistent to some degree with the data‐generating mechanism postulated by the sensitivity analysis assumption. Nevertheless, a strong advantage of such sensitivity analysis is the avoidance of full modelling under various, potentially very complex, missing data assumptions. However, when performing class 1 sensitivity analyses, the properties of an estimator under the primary analysis may change as we move to the sensitivity analysis. In particular, we shall see that a sensible variance estimator for the primary analysis may behave in an unexpected way under certain sensitivity analysis scenarios, e.g. decreasing as the proportion of missing values increases. In regulatory work, particularly in class 1 sensitivity analyses, it is therefore important to appreciate fully the quantity and nature of any additional statistical information about the treatment estimate that may arise in the sensitivity analysis, relative to the primary analysis.

This superficially abstract point can be readily illustrated. Suppose that a study intends to take measurements on *n* patients *Y*_1_,…,*Y*_*n*_, from a population with known variance *σ* ^2^, and the estimator is the mean. If no data are missing, then the statistical information about the mean is *n*/*σ* ^2^. Now suppose that a total of *n*_m_ observations are missing. We shall perform a class 1 sensitivity analysis, so that the estimator is the mean for both our primary and sensitivity analysis. Our primary analysis will assume that data are missing completely at random, and our sensitivity analysis will assume that the missing values are from patients with the same mean, but a different variance, $\sigma_{m}^{2}$.

Under our primary analysis assumption, we can obtain valid inference by calculating the mean of the *n*−*n*_m_ observed values, or by using multiple imputation for the missing values. In both cases the information about the mean is the same: (*n*−*n*_m_)/*σ* ^2^.

Under our class 1 sensitivity analysis, we multiply impute the missing data under our assumption, and again our estimator is the mean. Now, however, the statistical information will be approximately $n^{2}/\left\{\left(n-n_{m}\right)\sigma^{2}+n_{m}\sigma_{m}^{2}\right\}$. Further, the information about the mean from the sensitivity analysis depends on $\sigma_{m}^{2}$. Since $\sigma_{m}^{2}$ is not estimable, this information is under the control of the analyst.

**Figure 1:**
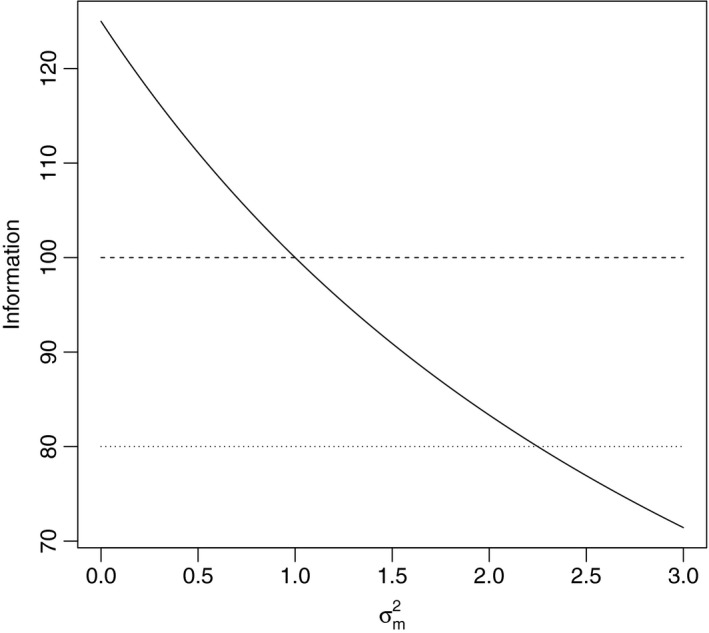
Information about the sample mean varies with $\sigma_{m}^{2}$: 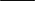, sensitivity analysis information; 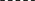, full data information; 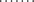, observed data information

This is illustrated by Fig. 1, which shows how the information about the mean varies with $\sigma_{m}^{2}$, when *n*=100, *n*_m_=20 and *σ* ^2^=1. When $\sigma_{m}^{2}<\sigma^{2},$ the information about the mean in the sensitivity analysis is greater than from the intended 100 observations; when $1\;⩽\;\sigma_{m}^{2}\;⩽\;2.25$ then the information is greater than in the *n*−*n*_m_ observations that we could obtain and, when $\sigma_{m}^{2}>2.25,$ the information is less than in the observed data (*n*−*n*_m_) observations we could obtain.

We believe that the International Conference on Harmonisation E9 (R1) addendum (2017) will lead to sensitivity analysis playing a much more central role; in this context we believe it important for statisticians and regulators to be aware of how—compared with the primary analysis—information can be removed or added in the sensitivity analysis.

Our purpose in this paper is

- to consider the information in sensitivity analyses, arguing that sensitivity analysis in a clinical trial should be information anchored—as defined below—relative to the primary analysis, and
- to demonstrate that using reference‐ and *δ*‐based controlled multiple imputation, with Rubin's rules, to perform class 1 sensitivity analyses is information anchored.

An important practical consequence of our work is that it provides a set of conditions that can be imposed on class 1 sensitivity analyses to ensure that—relative to the primary analysis—they neither create nor destroy statistical information. We believe that this provides important reassurance for their use, for example, in the regulatory setting.

The plan for the rest of the paper is as follows. Section 2 defines the concept of information anchoring in sensitivity analysis. Section 3 considers class 1 sensitivity analysis by reference‐ and *δ*‐based controlled multiple imputation, and presents our main theoretical results on information anchoring within this setting. Section 4 briefly reviews class 2 sensitivity analyses from this perspective. In Section 5 we present a simulation study which illustrates our theory for information‐anchored sensitivity analysis, which is then applied to a trial of training for peer reviewers in Section 6. We conclude with a discussion in Section 7.

The data that are analysed in the paper and the programs that were used to analyse them can be obtained from

https://rss.onlinelibrary.wiley.com/hub/journal/1467985x/seriesadatasets

## 2 Information‐anchored sensitivity analysis

We have seen in the simple example above how a sensitivity analysis can change the statistical information about a treatment estimate. We now define *information‐anchored* sensitivity analyses, which hold the proportion of information that is lost because of missing data constant across the primary and sensitivity analyses.

Suppose that a clinical trial intends to collect data from 2*n* patients, denoted **Y**, to estimate a treatment effect *θ*. However, a number of patients do not give complete data. Denote the observed data by **Y**_obs_, and missing data by **Y**_miss_. Consistent with the International Conference on Harmonisation E9 (R1) addendum (2017), we make a *primary* set of assumptions, under which we perform the primary analysis. We then make a *sensitivity* set of assumptions, under which we perform the sensitivity analysis. Both primary and sensitivity assumptions

- specify the distribution $\left[Y_{\mathrm{miss}}|Y_{\mathrm{obs}}\right]$,
- could be true, yet
- cannot be verified from **Y**_obs_.

Let ${\hat{\theta }}_{\mathrm{obs},\;\;\mathrm{primary}}$ be the estimate of *θ* under the primary analysis assumption. Further, suppose that we could observe a realization of **Y**_miss_ under the primary assumption. Putting these data together with **Y**_obs_ gives us a complete set of observed data, which actually follows the primary assumption: we denote this by **Y**_primary_, and the corresponding estimate of *θ* by ${\hat{\theta }}_{\mathrm{full},\;\;\mathrm{primary}}$. We denote the observed information about *θ* by $I({\hat{\theta }}_{\mathrm{obs},\;\;\mathrm{primary}})$ and $I({\hat{\theta }}_{\mathrm{full},\;\;\mathrm{primary}})$. Then,

$$\frac{I({\hat{\theta }}_{\mathrm{obs},\;\;\mathrm{primary}})}{I({\hat{\theta }}_{\mathrm{full},\;\;\mathrm{primary}})}<1,$$

reflecting the loss of information about *θ* due to missing data.

Defining corresponding quantities under the sensitivity assumptions for the chosen sensitivity analysis procedure (be this class 1 or class 2) we have

$$\frac{I({\hat{\theta }}_{\mathrm{obs},\;\;\mathrm{sensitivity}})}{I({\hat{\theta }}_{\mathrm{full},\;\;\mathrm{sensitivity}})}<1,$$

again reflecting the loss of information about *θ* due to missing data—but now under the sensitivity assumptions.

Comparing these leads us to the following definitions: information *negative* sensitivity analysis,

(1a) $$\frac{I({\hat{\theta }}_{\mathrm{obs},\;\;\mathrm{primary}})}{I({\hat{\theta }}_{\mathrm{full},\;\;\mathrm{primary}})}>\frac{I({\hat{\theta }}_{\mathrm{obs},\;\;\mathrm{sensitivity}})}{I({\hat{\theta }}_{\mathrm{full},\;\;\mathrm{sensitivity}})};$$

information‐*anchored* sensitivity analysis,

(1b) $$\frac{I({\hat{\theta }}_{\mathrm{obs},\;\;\mathrm{primary}})}{I({\hat{\theta }}_{\mathrm{full},\;\;\mathrm{primary}})}=\frac{I({\hat{\theta }}_{\mathrm{obs},\;\;\mathrm{sensitivity}})}{I({\hat{\theta }}_{\mathrm{full},\;\;\mathrm{sensitivity}})};$$

information *positive* sensitivity analysis,

(1c) $$\frac{I({\hat{\theta }}_{\mathrm{obs},\;\;\mathrm{primary}})}{I({\hat{\theta }}_{\mathrm{full},\;\;\mathrm{primary}})}<\frac{I({\hat{\theta }}_{\mathrm{obs},\;\;\mathrm{sensitivity}})}{I({\hat{\theta }}_{\mathrm{full},\;\;\mathrm{sensitivity}})}.$$

When analysing a clinical trial, we believe that an information positive sensitivity analysis is rarely justifiable, implying as it does that, the more data are missing, the more certain we are about the treatment effect under the sensitivity analysis. Conversely, although information negative sensitivity analyses provide an incentive for minimizing missing data, there is no natural consensus about the appropriate loss of information. Therefore, we argue that information‐anchored sensitivity analyses are the natural starting point. In regulatory work they provide an equal footing between regulators and industry, allowing the focus to be on the average response to treatment among the unobserved patients.

The definitions above are quite general, applying directly to class 1 and class 2 sensitivity analyses, and all types of *de jure* (on‐treatment) and *de facto* (as‐observed) assumptions. We now discuss class 1 sensitivity analyses from the information perspective and present our theory for information anchoring.

## 3 Class 1 sensitivity analysis and theory for information anchoring

Although class 1 sensitivity analyses can be performed without using multiple imputation (Lu, 2014; Liu and Pang, 2016; Tang, 2017), multiple imputation is the most flexible approach and often the simplest to implement (e.g. by using the SAS software from www.missingdata.org.uk or Stata software by Cro *et al*. (2016) or R code implementing related approaches by Moreno‐Betancur and Chavance (2016). This is generally called *controlled multiple imputation*, because the form of the imputation for the missing data is *controlled* by the analyst. So, for example, the analyst can control the imputed data mean to be *δ* below that under missingness at random (MAR). See, for example, Mallinckrodt (2013), chapter 10, O’Kelly and Ratitch (2014), pages 284–319, and Ayele *et al*. (2014).

One approach is to obtain information about parameters that control the departure from MAR from experts (Mason *et al*., 2017), but this is controversial (Heitjan, 2017), and challenging for longitudinal data where multiple parameters are involved. An alternative, as introduced by Little and Yau (1996) and developed and discussed further more recently by, among others, Carpenter *et al*. (2013), Ratitch *et al*. (2013) and Liu and Pang (2016), is *reference‐based* multiple imputation. In this approach, the distribution of the missing data is specified *by reference* to other groups of patients. This enables contextually relevant qualitative assumptions to be explored and avoids the need to specify numerical sensitivity parameters formally (these are implicit consequences of the appropriate reference for a patient). Some examples are listed in Table 1. For example, we may explore the consequences of patients in an active arm ‘jumping to reference’ post deviation. In practice the appropriate imputation model depends critically on the particular clinical setting and what assumptions are considered credible. Such analyses can be performed by using the reference‐based multiple‐imputation algorithm in the on‐line appendix A implemented in Cro *et al*. (2016). Overall, this approach is both very flexible and accessible, since patients’ missing outcomes are specified qualitatively—by reference to other groups of patients in the study. This explains its increasing popularity (Philipsen *et al*., 2015; Jans *et al*., 2015; Billings *et al*., 2018; Atri *et al*., 2018).

**Table 1:** Examples of reference‐based and external‐information‐controlled multiple‐imputation methods

*Name*	*Description*
*Reference‐based controlled multiple‐imputation methods*	
Jump to reference	Imputes assuming that following dropout a patient's mean profile follows that observed in the reference arm Pre‐dropout means come from the randomized arm
Copy increments in reference	Forms post‐dropout means by copying increments in the reference arm Pre‐dropout means come from the randomized arm
Last mean carried forward	Forms post‐dropout means by carrying forward the randomized arm mean at dropout
Copy reference	The conditional profile given the history is copied from the reference group, i.e. imputes as if randomized to the reference arm pre‐ and post‐dropout means come from the reference arm
*External‐information‐controlled multiple‐imputation methods*	
*δ*‐method	Impute under randomized arm MAR and subtract or add by fixed *δ*

These references all focus on clinical trials with continuous outcome measures that are collected longitudinally, and modelled using the multivariate normal distribution. We consider the same setting, and give criteria for class 1 sensitivity analysis using controlled multiple imputation with Rubin's variance formula to be information anchored. This shows that most forms of *δ*‐ and reference‐based imputation proposed in the literature are, to a good approximation, information anchored. It also shows that, in class 1 settings, uncritical use of the conventional primary analysis variance estimator is often information positive, which is undesirable in practice.

There are two principal reasons for this. The first is that class 1 sensitivity analyses retain the primary analysis model in the sensitivity analysis. However, in the sensitivity analysis, data assumptions are not wholly compatible with those of the primary analysis model. In particular variance estimators may behave in unexpected ways. The second reason is that reference‐based methods essentially use the data twice, e.g. by using data from the reference arm

- to impute missing data in an active arm and
- to estimate the effect of treatment in the reference arm.

### 3.1 Theoretical results

The presentation of our theoretical results is structured as follows. We begin by describing our data, model, primary analysis and sensitivity analysis. We show in corollary 2 that, when all data can be fully observed, for our treatment estimate $\hat{\theta },$

$$E\left[{\hat{V}}_{\mathrm{full},\;\;\mathrm{sensitivity}}\right]=E\left[{\hat{V}}_{\mathrm{full},\;\;\mathrm{primary}}\right]+O\left(n^{-2}\right).$$

Theorem Theorem 1 then defines the information‐anchored variance and derives a general expression for the difference between this and the variance from Rubin's rules. Finally, we show, in the remarks following theorem 1, that in practice this difference is small.

#### 3.1.1 Trial data

Consider a two‐arm trial, which includes *n* patients randomized to an active arm and *n* patients randomized to a reference arm (total 2*n* patients within the trial). Outcome data are recorded at *j*=1,...,*J* visits, where visit *j*=1 is the baseline. For patient *i* in treatment arm *z*, where *z*=*a* indicates active arm assignment and *z*=*r* indicates reference arm assignment, let *Y*_*z*,*i*,*j*_ denote the outcome at time *j*.

We wish to estimate the treatment effect at the end of the follow‐up, time *J*. Our analysis model is the regression of the outcome at time *J* on treatment and baseline (i.e. analysis of covariance). Now suppose that some patients are lost to follow‐up in the active arm (for simplicity, we assume for now that the reference arm data are complete). Our primary assumption is MAR.

Our primary analysis uses all the observed values, imputes the missing data under MAR, fits the analysis‐of‐covariance model to each imputed data set and combines the results using Rubin's rules (this is essentially equivalent to fitting a mixed model with unstructured mean and covariance matrix to the observed values; see Carpenter and Kenward (2008), chapter 3).

Our sensitivity analysis uses controlled multiple imputation, as formally defined below. This could include a *δ*‐based method or one of the reference‐based methods that are given in Table 1; all reference‐based multiple‐imputation methods can be implemented by using the generic algorithm in the on‐line appendix A.

For each trial arm, we assume a multivariate normal model, with common covariance matrix, so for patient *i* who has no missing values:

$$\left(\begin{array}{c} Y_{z,\;i,\;1}\\ Y_{z,\;i,\;2}\\ \vdots \\ Y_{z,\;i,\;J} \end{array}\right)∼N\left\{\left(\begin{array}{c} \mu_{z,\;1}\\ \mu_{z,\;2}\\ \vdots \\ \mu_{z,\;J} \end{array}\right)\;\;,\Sigma =\left(\begin{array}{cccc} \sigma_{1,\;1}^{2} & \sigma_{1,\;2}^{2} & \ldots & \sigma_{1,\;J}^{2}\\ \sigma_{1,\;2}^{2} & \sigma_{2,\;2}^{2} & \ldots & \sigma_{2,\;J}^{2}\\ \vdots & \vdots & \ldots & \vdots \\ \sigma_{1,\;J}^{2} & \sigma_{2,\;J}^{2} & \ldots & \sigma_{J,\;J}^{2} \end{array}\right)\right\}\;\;,$$

where *z*=*a* for the active patients and *z*=*r* for the reference patients.

Now suppose that all reference group patients and *n*_*o*_ active group patients follow the protocol, but *n*_*d*_=*n*−*n*_*o*_ active patients deviate from the protocol. Suppose that it was possible to continue to observe these *n*_*d*_ patients, but now their post‐deviation data follow the controlled model:

(2) $$\left(\begin{array}{c} Y_{a,\;i,\;1}\\ \vdots \\ Y_{a,\;i,\;j-1}\\ Y_{a,\;i,\;j}\\ Y_{a,\;i,\;j+1}\\ \vdots \\ Y_{a,\;i,\;J} \end{array}\right)∼N\left\{\left(\begin{array}{c} \mu_{a,\;1}\\ \vdots \\ \mu_{a,\;j-1}\\ \mu_{d,\;j,\;j}\\ \mu_{d,\;j,\;j+1}\\ \vdots \\ \mu_{d,\;j,\;J} \end{array}\right)\;\;,\Sigma \right\}\;\;.$$

The term ‘controlled’ means that the analyst controls the post‐deviation distribution. Here, for patient *i*, the first index indicates active or deviation, the second the time of deviation and the third the visit number. Different patients can deviate at different times, and this general formulation allows the pattern of their post‐deviation means to differ depending on their deviation time. This encompasses all the settings in Table 1, and others besides.

To present the theory, we first consider the case where the primary analysis does not adjust for baseline, extending to the baseline‐adjusted case in corollary 2.

Proposition 1 For the trial data that were described above, when the analysis model is a difference in means at the final time point with the usual sample variance estimate in both observed and controlled settings, then:

- if all patients follow the protocol and no data are missing, then the expectation of the variance estimate is
  $$E\left[{\hat{V}}_{\mathrm{full},\;\;\mathrm{primary}}\right]=\frac{2\sigma_{J,\;J}^{2}}{n};$$
- if *n*_*d*_ patients deviate and are observed following the controlled model (2) the expectation of the variance estimate is
  $$E\left[{\hat{V}}_{\mathrm{full},\;\;\mathrm{sensitivity}}\right]=\frac{2\sigma_{J,\;J}^{2}}{n}+\sum_{j=2}^{J}\frac{n_{o}n_{d,\;j}\Delta_{d,\;j}^{2}}{n^{3}}+\sum_{p=2}^{J}\overset{J}{\sum_{q=2}^{q\neq p}}\frac{n_{d,\;p}n_{d,\;q}\Delta_{d,\;p,\;q}^{2}}{n^{3}},$$
  where Δ_*d*,*j*_=*μ*_*a*,*J*_−*μ*_*d*,*j*,*J*_, Δ_*d*,*p*,*q*_=*μ*_*d*,*p*,*J*_−*μ*_*d*,*q*,*J*_ and we let *n*−1→*n*.

For a proof, see the on‐line appendix B.1.

Corollary Corollary 1 For clinical trials designed to detect a difference of *μ*_*a*,*J*_−*μ*_*r*,*J*_=Δ, with a significance level of *α* and power *β*, at the final visit *J*,

$$E\left[{\hat{V}}_{\mathrm{full},\;\;\mathrm{sensitivity}}\right]=E\left[{\hat{V}}_{\mathrm{full},\;\;\mathrm{primary}}\right]+O\left(n^{-2}\right).$$

First note that the standard sample size formula implies that

$$\Delta^{2}=\frac{2f\left(\alpha ,\beta \right)\sigma^{2}}{n}.$$

Therefore, Δ^2^ is *O*(*n* ^−1^). Further, since, in any trial, all $\Delta_{d,\;p,\;j}^{2}$ can be written as $\Delta_{d,\;p,\;j}^{2}=\kappa_{d,\;p,\;j}\Delta^{2}$ for some constant *κ*_*d*,*p*,*j*_, we have $\Delta_{d,\;p,\;j}^{2}=O\left(n^{-1}\right)$. Following the same arguments, $\Delta_{d,\;j}^{2}=O\left(n^{-1}\right)$. Second, note that *n*_*o*_/*n* is the proportion of active patients who complete the trial, and *n*_*d*,*j*_/*n* is the proportion who deviate at time *j*. Therefore, *n*_*on*__*d*,*j*_/*n* ^2^<1. Similarly *n*_*d*,*p*_ *n*_*d*,*q*_/*n* ^2^<1. It therefore follows that

(3) $$E\left[{\hat{V}}_{\mathrm{full},\;\;\mathrm{sensitivity}}\right]=E\left[{\hat{V}}_{\mathrm{full},\;\;\mathrm{primary}}\right]+O\left(n^{-2}\right).$$

Corollary Corollary 2 Under the conditions of corollary 1, if the primary analysis model is a linear regression of the outcome at the final time point, adjusted for baseline, then result (3) still holds.

Replace the unconditional variance $\sigma_{J,\;J}^{2}$ with the variance conditional on baseline, $\sigma_{J.1}^{2}=\sigma_{J,\;J}^{2}-{\left(\sigma_{1,\;J}^{2}\right)}^{2}/\sigma_{1,\;1}^{2}$, in the proof of proposition 1.

We now use this result in the context of reference‐based multiple imputation to calculate the difference between our defined information‐anchored variance and Rubin's multiple‐imputation variance.

Theorem Theorem 1 Consider a two‐arm trial which includes *n* patients randomized to an active arm and *n* patients randomized to a reference arm. Measurement data are recorded at *j*=1,…,*J* visits (where visit 1 is baseline). The primary analysis model is a linear regression of the outcome at the final time point (visit *J*) on baseline outcome and treatment. Suppose that all *n* of the reference arm are completely observed on reference treatment over the full duration of the trial (at all *J* visits) but, in the active arm, only *n*_*o*_ are observed without deviation. The remaining *n*_*d*_ patients in the active arm deviate at some point during the trial post baseline in a monotone fashion (such that *n*_*o*_+*n*_*d*_=*n*). Specifically, we assume that a proportion *π*_*d*,*j*_=*n*_*d*,*j*_/*n* drop out at each visit, for *j*>1, and their data are missing post deviation.

Assume that the primary design‐based analysis model satisfies result (3), and that the variance–covariance matrix for the data is the same in each arm. For each deviation pattern in the active arm who deviate at time *j*, let ${\overset{¯}{P}}_{a,\;d,\;j}$ be the *j*×1 mean vector of the *n*_*d*,*j*_ responses at times 1,…,*j*−1 plus a 1 (to allow for an intercept in the imputation model).

Suppose that the primary analysis is performed by multiple imputation assuming within‐arm MAR. Let ${\hat{V}}_{\mathrm{obs},\;\;\mathrm{primary}}$ denote the estimated variance for the treatment effect under the primary MAR assumption. Subsequently we perform class 1 sensitivity analysis via reference‐based multiple imputation, i.e. under equation (2), using the imputation algorithm in the on‐line appendix A. This general formulation includes all the reference‐based options in Table 1. As we are doing class 1 sensitivity analysis, the primary analysis model is used to analyse the imputed data. Then the difference between the information‐anchored variance of the sensitivity analysis treatment estimate, which is denoted by ${\hat{V}}_{\mathrm{anchored}}$, which by definition is $\left({\hat{V}}_{\mathrm{obs},\;\;\mathrm{primary}}/{\hat{V}}_{\mathrm{full},\;\;\mathrm{primary}}\right){\hat{V}}_{\mathrm{full},\;\;\mathrm{sensitivity}}$ and Rubin's multiple‐imputation variance, denoted by ${\hat{V}}_{{\mathrm{Rubin}}^{\prime }s\;\;\mathrm{MI}}$, is

(4) $$\begin{array}{cc} E\left[{\hat{V}}_{\mathrm{anchored}}\right]-E\left[{\hat{V}}_{{\mathrm{Rubin}}^{\prime }s\;\mathrm{MI}}\right] & =\sum_{j=2}^{J}\pi_{d,\;j}^{2}{\overset{¯}{P}}_{a,\;d,\;j}\left(V_{\mathrm{primary},\;j}-V_{\mathrm{sensitivity},\;j}\right){\overset{¯}{P}}_{a,\;d,\;j}^{T}\\ & \;+\frac{E[{\hat{B}}_{\mathrm{primary}}]}{O\left(n^{2}\right)E\left[{\hat{W}}_{\mathrm{primary}}\right]}. \end{array}$$

Here **V**_primary,*j*_ is the variance–covariance matrix of the parameter estimates in the primary MAR imputation model for deviation at time *j* and **V**_sensitivity,*j*_ is the variance–covariance matrix of the parameter estimates in the imputation model for deviation at time *j*, defined by the reference‐based sensitivity analysis assumption. ${\hat{B}}_{\mathrm{primary}}$ is the between‐imputation variance and ${\hat{W}}_{\mathrm{primary}}$ is the within‐imputation variance of the treatment effect in the primary analysis, both under MAR.

For a proof see the on‐line appendix B.2.

Theorem Theorem 1 establishes the difference between the information‐anchored variance and Rubin's rules variance. To show that class 1 sensitivity analysis by reference‐based multiple imputation is information anchored, we need to consider how close expression (4) is to 0.

The key quantity driving the approximation is the first of the two terms. Note that, for each deviation time *j*, the variance–covariance matrix of the parameters of the on‐treatment imputation model is **V**_primary,*j*_=**Σ**_*j*_/*n*_*o*_, where **Σ**_*j*_ is the relevant submatrix of the variance–covariance matrix **Σ** of the *J* observations. The precise form of **V**_sensitivity,*j*_ will depend on the sensitivity analysis imputation model. Consider that data from the fully observed reference arm are used in the sensitivity imputation (e.g. copy reference). In this case, **V**_sensitivity,*j*_=**Σ**_*j*_/*n*, and

$$\begin{array}{cc} \pi_{d,\;j}^{2}{\overset{¯}{P}}_{a,\;d,\;j}\left(V_{\mathrm{primary},\;j}-V_{\mathrm{sensitivity},\;j}\right){\overset{¯}{P}}_{a,\;d,\;j}^{T} & =\pi_{d,\;j}^{2}{\overset{¯}{P}}_{a,\;d,\;j}\Sigma_{j}(\frac{1}{n_{o}}-\frac{1}{n}){\overset{¯}{P}}_{a,\;d,\;j}^{T}\\ & =\pi_{d,\;j}^{2}{\overset{¯}{P}}_{a,\;d,\;j}\Sigma_{j}\frac{n-n_{o}}{n_{o}n}{\overset{¯}{P}}_{a,\;d,\;j}^{T}\\ & =\pi_{d,\;j}^{2}{\overset{¯}{P}}_{a,\;d,\;j}\Sigma_{j}\frac{\pi_{d}}{n(1-\pi_{d})}{\overset{¯}{P}}_{a,\;d,\;j}^{T}. \end{array}$$

Applying this line of argument to the other methods in Table 1 suggests that the error in the approximation will be small and will vanish asymptotically.

Thus we have established that class 1 referenced‐based imputation sensitivity analysis is, to a good approximation, information anchored. We illustrate this in the simulation study in Section 5.

### 3.2 Further comments

- In the proof of theorem 1, to simplify the argument, the variance–covariance matrix of the data **Σ** is assumed known *in the imputation model*. When—as will generally be so—it must be estimated, Carpenter and Kenward (2013), pages 58–59, show that, for the simple case of the sample mean, the additional bias is small and vanishes asymptotically. This strongly suggests that any additional bias caused by estimating the variance–covariance matrix will be small, and asymptotically irrelevant; this is borne out by our simulation studies below.
- For simplicity the theory treated the deviation pattern as fixed. We can replace all the proportions *π*_*d*,*j*_ by their sample estimates and then take expectations over these in a further stage. As our results are asymptotic, the conclusions will be asymptotically equivalent.
- *δ*‐method sensitivity analysis: we consider that, at the final time point *J*, imputed values for patients who deviate at time *j* (for *j*>1) are edited by (*J*+1−*j*)*δ* to represent a change in the rate of response of *δ* per time point post deviation. We now evaluate the size of the two terms in expression (4) separately. For the first term, when *δ* is fixed, the covariance matrix for the imputation coefficients under the primary analysis and the sensitivity analysis is identical for each missing data pattern *j*; the *δ*‐method simply adds a constant to the imputed values. Consequently **V**_primary,*j*_=**V**_sensitivity,*j*_; thus $\pi_{d,\;j}^{2}{\overset{¯}{P}}_{a,\;d,\;j}\left(V_{\mathrm{primary},\;j}-V_{\mathrm{sensitivity},\;j}\right){\overset{¯}{P}}_{a,\;d,\;j}^{T}=0,$ and Rubin's rules give a very sharp approximation to the information‐anchored variance.
  However, when *δ* is not fixed and we vary *δ* over the imputation set *K*, i.e. we suppose that $\delta_{k}∼N\left(\delta ,\sigma_{\delta }^{2}\right)$, then, $\pi_{d,\;j}^{2}{\overset{¯}{P}}_{a,\;d,\;j}\left(V_{\mathrm{primary},\;j}-V_{\mathrm{sensitivity},\;j}\right){\overset{¯}{P}}_{a,\;d,\;j}^{T}=-\pi_{d}^{2}\sigma_{\delta }^{2}$, and the sensitivity analysis is *information negative*. The extent of this is principally driven by the variance of *δ*_*k*_.
  Now consider the second term in expression (4). When the *δ*‐method is used it is not necessarily the case that result (3) holds, since Δ_*d*,*j*_=*μ*_*a*,*J*_−*μ*_*d*,*j*,*J*_ and Δ_*d*,*p*,*q*_=*μ*_*d*,*p*,*J*_−*μ*_*d*,*q*,*J*_ are not necessarily *O*(*n* ^−1^). In the *δ*‐based scenario, as outlined in the on‐line appendix B.1,
  $${\hat{V}}_{\mathrm{full},\;\;\mathrm{sensitivity}}={\hat{V}}_{\mathrm{full},\;\;\mathrm{primary}}+Q,$$
  where
  $$Q=\sum_{j=2}^{J}\frac{n_{o}n_{d,\;j}{\left(J+1-j\right)}^{2}\delta^{2}}{n^{3}}+\sum_{p=2}^{J}\sum_{q=2}^{J,\;q\neq p}\frac{n_{d,\;p}n_{d,\;q}{\left\{\left(J+1-p\right)\delta -\left(J+1-q\right)\delta \right\}}^{2}}{n^{3}}.$$
  Thus, for the *δ*‐method the *O*(*n* ^−2^) component in the second term of expression (4) is replaced with *Q* (as defined above). The composition of *Q* indicates that the information anchoring performance of Rubin's variance estimate will also depend on the size of *δ*. Typically, the size of *δ* will not have a large effect since the terms in *Q* are all multiplied by components of the form *n*_*on*__*d*,*j*_/*n* ^3^ or *n*_*d*,*p*_ *n*_*d*,*q*_/*n* ^3^ and thus will vanish asymptotically. Hence with a fixed *δ* adjustment, the information anchoring approximation will typically be excellent.
- Improved information anchoring: remark (b) shows that, provided that the underlying variance–covariance matrices of the data are similar, the key error term in the information anchoring approximation is the difference in precision with which they are estimated. If all *n* patients are observed in the reference arm and *n*_*o*_ in the active arm, this is
  $$\frac{1}{n_{o}}-\frac{1}{n}.$$
  This suggests that Rubin's rules will lead to improved information anchoring if, instead of using all patients in the reference arm to estimate the imputation model for deviators at time *j*, a random *n*_*o*_ are used. We have confirmed this by simulation, but the improvement is negligible when the proportion of missing data is less than 40%, when simulations confirm that the approximation is typically excellent.
- Theorem Theorem 1 suggests that, for a given deviation pattern, information anchoring will be worse the greater the difference between the covariance matrix of the imputation coefficients under the primary and sensitivity analysis. However, we have not encountered examples where this has been a practical concern.
- We have not presented formal extensions of our theory to the case when we also have missing data in the reference arm. But this does not introduce any substantial errors in the information anchoring approximation. With missing data in the reference arm, for each missing data pattern *j*, an additional component which depends on the difference between the variance of the imputation parameters in the primary on‐treatment imputation model and sensitivity scenario imputation model for the reference arm, multiplied by the proportion of reference patients with that missing data pattern squared (denoted $\pi_{r,\;d,\;j}^{2}$), is included. If reference arm data are imputed under within‐arm MAR (as under copy increments in reference, copy reference or jump to reference) these terms will be 0. In the more general case, where different patterns of patients, across different arms, are imputed with different reference‐based assumptions, additional non‐zero error terms of the form as in the summation in expression (4) will be introduced; but again, for the reasons discussed above, these will typically be small. The covariance between the parameters of the active and reference arm sensitivity scenario imputation models for each missing data pattern also contributes to the sharpness of the approximation. The exact size of these additional error terms again depends on the specific sensitivity scenario and in some cases will be 0 (e.g. last mean carried forward). But each covariance term is always multiplied by the proportion of deviators in each arm with the associated missing data patterns (*π*_*d*,*j*_ *π*_*r*,*d*,*j*_), ${\overset{¯}{P}}_{a,\;d,\;j}$ and ${\overset{¯}{P}}_{r,\;d,\;j}^{T}$ (the *j*×1 mean vector of the responses at times 1,…,*j*−1 for the reference patients deviating at time *j*, plus a 1 to allow for an intercept in the imputation model). Thus it will be of a relatively small order in practice following the reasons that were discussed above.

#### 3.2.1 Summarizing remark

Given a primary design‐based analysis model, we have established in proposition 1 a criterion which defines a general class of reference‐based sensitivity analyses. If these sensitivity analyses are performed by multiple imputation, we have further established in theorem 1 that they will be—to a good approximation—information anchored, in line with the principles that we set out in Section 2. We have also shown why the information anchoring is particularly sharp for the *δ*‐method of multiple imputation.

## 4 Class 2 sensitivity analyses and information anchoring

A full exploration of information anchoring for class 2 sensitivity analyses is beyond the scope of this paper. Here, we focus on likelihood‐based selection models (see, for example, Diggle and Kenward (1994)) and use the results of Molenberghs *et al*. (1998) to make links to pattern mixture models, which enables us to use the results that we presented in Section 3.

Continuing with the setting in Section 4, consider a trial with scheduled measurement times of a continuous outcome measure at baseline and over the course of the follow‐up. When data are complete, the primary analysis is the analysis of covariance of the outcome measure at the scheduled end of follow‐up on baseline and treatment group. Equivalent estimates and inferences can be obtained from a mixed model fitted to all the observed data, provided that we have a common unstructured covariance matrix and a full treatment–time and baseline–time interaction.

Now suppose that patients withdraw before the scheduled end of follow‐up and subsequent data are missing. The mixed model that was described in the previous paragraph then provides valid inference under the assumption that post‐withdrawal data are missing at random given baseline, treatment group and available follow‐up data. A selection model that allows post‐withdrawal data to be missing not at random combines this mixed model with a model for the dropout process. Let *R*_*i*,*j*_ equal 1 or 0 if we respectively observe or miss the outcome for patient *i* at scheduled visit *j*=1,…,*J*. An illustrative selection model is

(5) $$\begin{array}{cc} Y_{i,\;j} & =\alpha_{j}+\beta_{j}Y_{i,\;0}+\gamma_{j}T_{i}+\epsilon_{i,\;j},\epsilon_{i}∼N\left(0,\Sigma_{J\times J}\right),\\ g(R_{i,\;j}) & =\alpha_{j}^{R}+\beta_{j}^{R}Y_{i,\;0}+\gamma_{j}^{R}T_{i}+\delta_{1}^{R}Y_{i,\;j-1}+\delta_{2}^{R}\left(Y_{i,\;j}-Y_{i,\;j-1}\right), \end{array}$$

where the superscript *R* denotes a selection model parameter, and the link function *g* is typically the logit, probit or complementary log–log‐link (the last giving a discrete time proportional hazards model for withdrawal).

Usually there is little information on the informative missingness parameter $\delta_{2}^{R}$ in the data (Rotnitzky *et al*., 2000; Kenward, 1998), and this information will be highly dependent on the data distribution assumed. Therefore, in applications it is more useful to explore the robustness of inferences to specific, fixed, values of $\delta_{2}^{R}$ ($\delta_{2}^{R}=0$ corresponds to MAR).

For each of these specific values of $\delta_{2}^{R},$ we may recast the selection model as a pattern mixture model, following Molenberghs *et al*. (1998). The differences between the observed and unobserved patterns are defined as functions of the fixed $\delta_{2}^{R}$. However, these then become a particular example of the *δ*‐method pattern mixture models that were considered in Section 3, which we have shown are information anchoring.

More generally, local departures from MAR are asymptotically information anchored. To see this, denote by ***θ*** the parameters in equation (5), apart from $\delta_{2}^{R}$. For a fixed $\delta_{2}^{R},$ let $i(\hat{\theta };\delta_{2}^{R})$ be the observed information matrix at the corresponding maximum likelihood estimates $\hat{\theta }$. For regular log‐likelihoods and a given data set, as we move away from MAR, for each element *i* of the information matrix **i**, the mean value theorem gives

(6) $$i\left(\hat{\theta };\delta_{2}^{R}\right)-i\left(\hat{\theta };0\right)=\left\{\frac{\partial }{\partial \delta_{2}^{R}}i\left(\hat{\theta };\delta_{2}^{R}\right){|}_{\delta_{2}^{R}={\tilde{\delta }}_{2}^{R}}\right\}\delta_{2}^{R},\text{for some}\;{\tilde{\delta }}_{2}^{R}\in \left(0,\delta_{2}^{R}\right).$$

However, asymptotically the parameter estimates are normally distributed, so the third derivative of the likelihood (i.e. the right‐hand side of expression (6)) goes to 0. Because result (6) holds when we use both the full data, and the partially observed data, it is sufficient to give information anchoring. This is the basis for our intuition that, for most phase III trials, class 2 sensitivity analyses can be treated as information anchored for practical purposes.

## 5 Simulation study

We now present a simulation study which illustrates the information anchoring property of Rubin's variance formula, derived in Section 3. The simulation study is based on a double‐blind chronic asthma randomized controlled trial that was conducted by Busse *et al*. (1998). The trial compared four doses of the active treatment budesonide against placebo on forced expiratory volume FEV_1_ (recorded in litres) over a period of 12 weeks. FEV_1_‐measurements were recorded at baseline and after 2, 4, 8 and 12 weeks of treatment. The trial was designed to have 80% power (5% type 1 error) to detect a change of 0.23 in FEV_1_ with 75 patients per arm, assuming a standard deviation SD of 0.5.

We simulated longitudinal data, consisting of baseline (time 1) and two follow‐up time points (time 2 being week 4, and time 3 being week 12), from a multivariate normal distribution whose mean and covariance matrix were similar to those observed in the placebo and lowest active dose arm of this trial:

$$\begin{array}{cc} & \Sigma_{\mathrm{placebo}}=\Sigma_{\mathrm{active}}=\left(\begin{array}{ccc} 0.4 & 0.2 & 0.2\\ 0.2 & 0.5 & 0.2\\ 0.2 & 0.2 & 0.6 \end{array}\right)\;\;,\\ & \mu_{\mathrm{placebo}}=\left(2.0,1.95,1.9\right),\\ & \mu_{\mathrm{active}}=\left(2.0,2.21,\mu_{a,\;3}\right) \end{array}$$

(litres).

In the asthma study *μ*_*a*,3_≈2.2, corresponding to a treatment effect of approximately 0.3 at time 3 (week 12). In the simulation study we explored *μ*_*a*,3_={1.9,2.2,2.9}. To test approximation (4) we chose a sample size of *n*=250 in each arm, giving a power of at least 90% in all scenarios. For each scenario, the analysis model was a linear regression of FEV_1_ at time 3 on baseline and treatment, and this was fitted to the full data.

Subsequently, for the active arm, we simulated monotone deviation completely at random. We varied the proportion of patients deviating overall from 0% to 50%. For each overall proportion deviating, around half the patients deviated completely at random before time 2, and around half deviated completely at random before time 3. All post‐deviation data were set to missing. The reference arm was always fully observed.

For each simulated data set, the primary analysis assumed MAR, and we performed class 1 sensitivity analyses using each of the reference‐based methods in Table 1 and Rubin's variance was calculated. 50 imputations were used for each analysis. For the *δ*‐method, the unobserved data were postulated to be worse (than under MAR) by a fixed amount of *δ*={0,−0.1,−0.5,−1}, for each time point post deviation, where *δ*=0 is equivalent to the primary, MAR, analysis. Thus, for patients who deviated between time 1 and 2, their missing at random imputed observations at time 2 were altered by *δ* and at time 3 by 2*δ*. For patients who deviated between time 2 and 3, their missing at random imputed observation at time 3 was altered by *δ*.

1000 independent replicates were generated for each combination of *μ*_*a*,3_ and deviation proportion. Our results focus on the time 3 treatment effect and its variance.

To minimize the Monte Carlo variability in our comparisons, we used the same set of 1000 data sets and deviation patterns for each sensitivity analysis.

Within each replication, for each sensitivity scenario, we also drew post‐deviation data under this scenario, giving a complete scenario‐specific data set. For each replication this allowed us to estimate the treatment effect and ${\hat{V}}_{\mathrm{full},\;\;\mathrm{sensitivity}}$ for each scenario. Then, we calculated the theoretical information‐anchored variance, which by definition in Section 2 is ${\hat{V}}_{\mathrm{anchored}}=\left({\hat{V}}_{\mathrm{obs},\;\;\mathrm{primary}}/{\hat{V}}_{\mathrm{full},\;\;\mathrm{primary}}\right){\hat{V}}_{\mathrm{full},\;\;\mathrm{sensitivity}}$. Estimates were averaged over the 1000 simulations. All simulations were performed using Stata version 14 (StataCorp, 2015) and reference‐based multiple imputation was conducted by using the mimix program by Cro *et al*. (2016).

### 5.1 Simulation results

**Figure 2:**
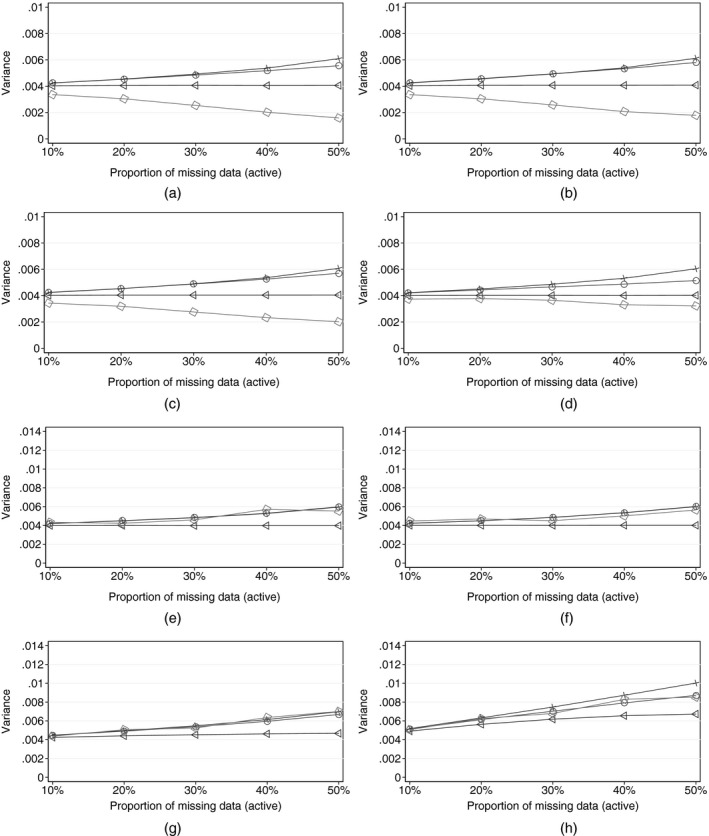
Simulation results (for each sensitivity scenario, as the proportion of active arm deviations increases, each panel shows the evolution of the mean estimate of the time 3 treatment effect variance (over 1000 replications) calculated in four ways: 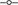, Rubin's multiple‐imputation variance, from reference‐ or *δ*‐based multiple imputation; 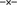, information‐anchored variance ($\hat{E}\left[{\hat{V}}_{\mathrm{anchored}}\right]$); 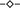, applying the primary analysis variance estimator in sensitivity scenarios; 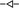, variance when post‐deviation data are actually fully observed under the given scenario ($\hat{E}\left[{\hat{V}}_{\mathrm{full},\;\mathrm{sensitivity}}\right]$)): (a) sensitivity scenario, copy reference; (b) sensitivity scenario, jump to reference; (c) sensitivity scenario, copy increments in reference; (d) sensitivity scenario, last mean carried forward; (e) sensitivity scenario, *δ*‐method, with *δ*=0 (MAR); (f) sensitivity scenario, *δ*‐method, with *δ*=−0.1; (g) sensitivity scenario, *δ*‐method, with *δ*=−0.5; (h) sensitivity scenario, *δ*‐method, with *δ*=−1.0

Fig. 2 shows the results, for each of the reference‐based sensitivity scenarios in Table 1, and controlled multiple imputation with four values of *δ*.

Figs 2(a)–2(d) display the results for the reference‐based scenarios for a moderate treatment effect of 0.3 (*μ*_*a*,3_=2.2), comparable with that found in the asthma trial. We see that the results show excellent information anchoring by Rubin's variance estimator for up to 40% of patients deviating. Note that the information‐anchored variance is always greater than ${\hat{V}}_{\mathrm{full},\;\;\mathrm{sensitivity}}$: the variance that we would see if we could observe data under the sensitivity assumption.

**Figure 3:**
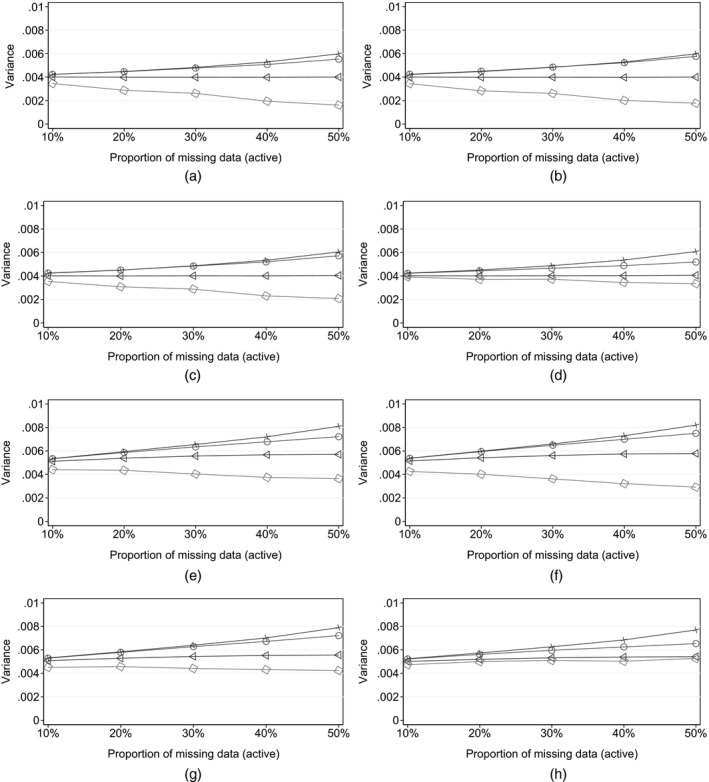
Simulation results (for each sensitivity scenario, as the proportion of active arm deviations increases, each panel shows the evolution of the mean estimate of the time 3 treatment effect variance (over 1000 simulations) calculated in four ways: 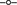, Rubin's multiple‐imputation variance, from reference‐ or *δ*‐based MI; 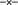, information‐anchored variance ($\hat{E}\left[{\hat{V}}_{\mathrm{anchored}}\right]$); 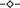, applying the primary analysis variance estimator in sensitivity scenarios; 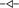, variance when post‐deviation data are alternatively fully observed under the given scenario ($\hat{E}\left[{\hat{V}}_{\mathrm{full},\;\mathrm{sensitivity}}\right]$)): (a) sensitivity scenario, copy reference, treatment effect 0; (b) sensitivity scenario, jump to reference, treatment effect 0; (c) sensitivity scenario, copy increments in reference, treatment effect 0; (d) sensitivity scenario, last mean carried forward, treatment effect 0; (e) sensitivity scenario, copy reference, treatment effect 1.0; (f) sensitivity scenario, jump to reference, treatment effect 1.0; (g) sensitivity scenario, copy increments in reference, treatment effect 1.0; (h) sensitivity scenario, last mean carried forward, treatment effect 1.0

These results are echoed by those with smaller and larger treatment effects (Fig. 3). We conclude that, for realistic proportions of missing post‐deviation data, reference‐based multiple imputation using Rubin's variance estimator can be regarded as information anchored.

This is in contrast with the behaviour of the conventional variance estimator from the primary regression analysis. Across all four reference‐based scenarios, this reduces—and tends to 0—as the proportion of missing data increases, and so yields increasingly information positive inference as more data are missing! It is also smaller than the variance that we would obtain *if we could observe data under the sensitivity assumption*. Therefore (see Carpenter *et al*. (2014)), we believe that this is not generally an appropriate variance estimator for class 1 sensitivity analyses. We return to this point below.

Now consider Figs 2(e)–2(h), which show results for controlled multiple imputation using the *δ*‐method. Again, consistent with the theory in Section 3, these show excellent information anchoring by Rubin's variance estimator for all missingness scenarios for *δ*=0,−0.1,−0.5, l. Indeed, the information anchoring approximation is better than for the reference‐based methods above because the covariance matrix for the imputation coefficients under MAR and *δ*‐based imputation are identical: the first term in expression (4) disappears.

For contextually large *δ*=−1 l, the approximation is excellent for up to 40% missing data. For greater proportions of missingness the approximation is not so sharp, and this is caused by the size of the second term in expression (4), which is larger with a bigger *δ* and greater proportion of missing post‐deviation data.

For the *δ*‐method we also see that using the conventional variance estimator from the primary analysis is also information anchored. The reason for different behaviour here from that for reference‐based methods is that reference‐based methods borrow information from another trial arm, and they do this increasingly as the proportion of patients deviating increases. This causes the conventional variance estimator to be information positive. However, with the*δ*‐method there is no borrowing between arms, so this issue does not arise.

To summarize, the simulations demonstrate our theoretical results, showing that, for all the controlled multiple‐imputation methods outlined in Table 1 (reference and *δ* based), in realistic trial settings multiple imputation using Rubin's rules gives information‐anchored inference for treatment effects. It is only with very high proportions of missing data (e.g. greater than 50%) that the information anchoring performance of Rubin's variance begins to deteriorate. Such high proportions of missing data are unlikely in well‐designed trials and would typically be indicative of other major problems.

## 6 Analysis of a peer review trial

We now illustrate how the information‐anchored theory that was outlined in Section 3 performs in practice, using data from a single‐blind randomized controlled trial of training methods for peer reviewers for the *British Medical Journal*. Full details of the trial were given in Schroter *et al*. (2004).

### 6.1 Description of the data

Following concerns about the quality of peer review, the original trial was set up to evaluate no training, face‐to‐face training or a self‐taught training package. After consent, but before randomization, each participant was sent a baseline manuscript to review (paper 1) and the quality of review was measured by using the review quality index (RQI). This is a validated instrument which contains eight items and is scored from 1 to 5, where a perfect review would score 5. All 609 participants who returned their review of paper 1 were randomized to receive one of the three interventions.

2–3 months later, participants were sent a further manuscript to review (paper 2). If this manuscript was reviewed a third was sent 3 months later (paper 3). Unfortunately, not all the reviewers completed the required reviews; thus some review scores were missing. The main trial analysis was conducted under the MAR assumption, using a linear regression of RQI on intervention group adjusted for baseline RQI. The analysis showed that the only statistically significant difference was in the quality of the review of paper 2, where the self‐taught group did significantly better than the no‐training group.

**Figure 4:**
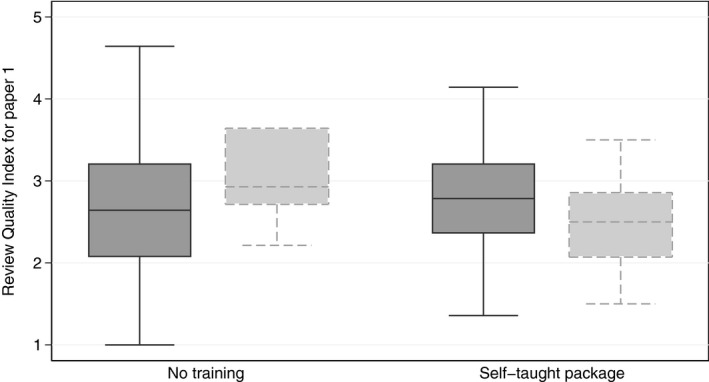
Quality of the baseline review: 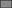, reviewed paper 2; 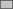, did not review paper 2

Therefore, here we focus on examining the robustness of this purportedly significant result to different assumptions about the missing data. Assuming MAR, the analysis found that reviewers in the self‐taught group had a mean RQI 0.237 points above the no‐intervention group (95% confidence interval 0.01–0.37; *p*=0.001). Although this is relatively small, the self‐taught intervention is inexpensive and may be worth pursuing. However, Fig. 4 shows the quality of the review at baseline for

- those who went on to complete the second review and
- those who did not,

for each of these two trial arms. The results suggest that a disproportionate number of poor reviewers in the self‐taught group failed to review paper 2. This suggests that the MAR assumption may be inappropriate, and data may be missing not at random.

### 6.2 Statistical analysis

The primary analysis model was a linear regression of paper 2 RQI on baseline and intervention group (self‐taught *versus* no training), and the intervention effect estimate is shown in the first row of Table 2.

**Table 2:** Estimated effect of self‐training *versus* no training on the paper 2 RQI, from the primary and various sensitivity analyses

*Analysis*	*Estimate*	*Standard error*	*p*‐*value*
Primary analysis, MAR	0.237	0.070	0.001
Multiple imputation, MAR	0.234	0.071	0.001
Multiple imputation, copy no training†	0.172	0.069	0.013
Multiple imputation, expert opinion	0.195	0.132	0.145
*δ*_*k*_∼*N*(−0.21,0.46^2^)			
Multiple imputation, *δ*‐method with	0.189	0.072	0.009
*δ*=−0.21†			

† Information‐anchored sensitivity analysis.

We conducted four further analyses.

- We multiply imputed the missing RQI data assuming MAR, fitted the primary analysis model to each imputed data set and combined the results for inference by using Rubin's rules. The imputation model for RQI of paper 2 included the variables that were present in the primary analysis model (RQI at baseline and treatment group).
- As it is reasonable to suppose that many of the reviewers in the self‐taught group who did not return their second review ignored their training materials, we perform a class 1 sensitivity analysis assuming that they ‘copied no training’. We used multiple imputation and Rubin's rules for information‐anchored inference.
- We reproduced a previous sensitivity analysis that was described by White *et al*. (2007). They used a questionnaire to elicit experts’ prior opinion about the average difference in RQI between those who did, and did not, return the review of paper 2 (20 editors and other staff at the *British Medical Journal* completed the questionnaire). The resulting distribution can be summarized as *N*(−0.21,0.46^2^). We used this to perform a *δ*‐method sensitivity analysis, where, for each imputation *k*, RQI values in the self‐taught arm were imputed under MAR and then had *δ*_*k*_∼*N*(−0.21,0.46^2^) added. This analysis is expected to be information negative.
- Our fourth analysis used the *δ*‐method via multiple imputation for participants in the self‐taught arm, but now fixed *δ*=−0.21 (the mean expert opinion) to obtain information‐anchored analysis.

All analyses used 50 imputations and were performed using Stata version 14 (StataCorp (2015)).

### 6.3 Results

Table 2 shows the results. As theory predicts, the first and second rows show that the primary analysis and analysis assuming MAR using multiple imputation give virtually identical results. In the third row, reference‐based sensitivity analysis assuming copy no training reduces the estimated effect to 0.172; compared with the primary analysis the information‐anchored standard error is now very slightly reduced at 0.069. The effect of this is to increase the *p*‐value by a factor of 10 to 0.013.

In contrast, using the expert's prior distribution (the fourth row), the point estimate is 0.195, but the standard error is much increased at 0.132, so the *p*‐value is over 100 times greater than in the primary analysis. Lastly (the fifth row), again using the *δ*‐method, but now fixing *δ*=−0.21, gives a similar point estimate, but an information‐anchored standard error of 0.072.

Critically, comparing the last two rows shows that expert opinion loses a further

$$\frac{1/0.072^{2}-1/0.132^{2}}{1/0.072^{2}}\times 100=70\%$$

of the information *beyond that lost due to missing data under the primary analysis*. Such information losses are not atypical (Mason *et al*., 2017). Since trials are often powered with minimal regard to potential missing data, such a loss of information must frequently lead to the primary analysis being overturned. By contrast, information‐anchored sensitivity analysis fixes the loss of information across the primary and sensitivity analysis, at a level that is possible to estimate *a priori* for any given deviation pattern.

## 7 Discussion

The recent publication of the International Conference on Harmonisation E9 (R1) addendum (2017) is bringing a sharper focus on the estimand. As the addendum recognizes, this in turn leads to a greater focus on the assumptions underpinning estimands. When we are faced with estimand relevant protocol deviations, or intercurrent events (e.g. rescue medication) and loss to follow‐up etc., such assumptions are at best only partially verifiable from the actual trial data. In such settings, a primary analysis assumption is made, and then the robustness of inferences to some secondary sensitivity assumptions will ideally be explored.

The assumptions underpinning the primary and sensitivity analyses should be as accessible as possible. This applies not only to assumptions about the typical, or mean, profile of patients post deviation, but also to assumptions about their precision.

In this paper, we have introduced the concept of *information anchoring*—whereby the extent of information loss due to missing data is held constant across primary and sensitivity analyses. We believe that this facilitates informed inferences and decisions, whatever statistical method is adopted. Information anchoring allows stakeholders to focus on the assumptions about the mean responses of each patient, or group of patients, post deviation, without being concerned about whether we are injecting information into or removing information from the analysis (relative to that lost—due to patient deviations—in the primary analysis). For example, we believe that this provides a good basis for discussions between regulators and pharmaceutical statisticians: the former can be reassured that the sensitivity analysis is not injecting information, whereas the latter can be reassured that the sensitivity analysis is not discarding information.

We have differentiated between two types of sensitivity analysis: class 1 and class 2. In class 1 the primary analysis model is retained in the sensitivity analysis; such sensitivity analyses can be readily (but need not be) carried out by multiple imputation.

Controlled multiple‐imputation procedures, which combine a pattern mixture modelling approach with multiple imputation, naturally fall into this first class. These include reference‐based multiple‐imputation procedures, which impute missing data under qualitative assumptions for the unobserved data, based on data observed in a specified reference group. The primary analysis model is retained in the sensitivity analyses, fitted to each imputed data set and results combined by using Rubin's rules. Consequently the assumptions of the primary analysis model are generally inconsistent with the data‐generating mechanism postulated by the sensitivity analysis assumption. Thus the usual justification for Rubin's multiple‐imputation rules does not hold. Instead, we have identified a new property of these rules, namely that for a broad class of controlled multiple‐imputation approaches, including both *δ*‐ and reference‐based approaches, they yield information‐anchored inference. In this regard, a practically important corollary of our theory is that the widely used *δ*‐method (and associated tipping point analysis) is information anchored with fixed *δ* adjustment.

Although we believe that information‐anchored sensitivity analyses provide a natural starting point and will often be sufficient, in certain scenarios it may also be desirable to conduct information negative sensitivity analysis. In such analyses a greater loss of information due to post‐deviation (missing) data is imposed by the analyst in the sensitivity analysis relative to the primary analysis. One way to do this is by prior elicitation—i.e. incorporating a prior distribution on *δ*—as touched on in the further comments following theorem 1 and Section 6. The theory in Section 3 also shows how a greater loss of information can be imposed in sensitivity analysis via reference‐based multiple imputation if required. This is done by reducing the size of the reference group that is used to construct the reference‐based imputation models.

Whatever approach is taken, careful thought needs to be given, and justification provided, for the additional loss of information being imposed. As we discussed at the end of Section 6, the loss of information with prior elicitation can be substantial. Often it will be difficult to justify an additional amount of information loss to impose.

Conversely, we argue that information positive sensitivity analysis, where a lower loss of information due to missing data post deviation is imposed in the sensitivity analysis relative to the primary analysis, is rarely justifiable, if at all. This is because it goes against all our intuition that missing data means that we lose (not gain) information: with information positive sensitivity analyses, we gain more precise inferences the more data we lose!

Our approach to determining the appropriate information in sensitivity analyses (which, as the simple example in Section 1 shows, is under the control of the analyst), contrasts with some recent work. Lu (2014), Tang (2017) and Liu and Pang (2016) each developed alternative implementations of the reference‐based pattern mixture modelling approach. Lu (2014) introduced an analytical approach for placebo‐based (copy reference) pattern mixture modelling which uses maximum likelihood and the delta method for treatment effect and variance estimation. Tang (2017) derived different analytical expressions for reference‐based models, also via the likelihood‐based approach. Liu and Pang (2016) proposed a Bayesian analysis for reference‐based methods which estimates the treatment effect and variance from the posterior distribution.

What Lu (2014), Liu and Pang (2016) and Tang (2017) have in common is that, in the terminology that is developed here, they essentially choose to apply the primary analysis variance estimator across the sensitivity analyses. Although this choice has a long‐run justification, for the reference‐based multiple‐imputation estimator, as our simulation results in Fig. 2 show (and we have discussed elsewhere (Carpenter *et al*., 2014)), this choice also means information positive inferences for reference‐based scenarios. This is a consequence of

- uncongeniality between the imputation and analysis model and
- the fact that reference‐based methods borrow information from within and across arms.

Thus we highlight here that, if one of these alternative implementations is employed within sensitivity analysis, information positive inference will be obtained.

What are the implications of this for our approach? Necessarily, the variance estimate arising from the information‐anchored sensitivity analysis via reference‐based multiple imputation does not have a long‐run justification for the reference‐based multiple‐imputation point estimate. However, having determined that the information‐anchored variance is appropriate, we can readily inflate the long‐run variance of the reference‐based multiple‐imputation estimator by adding appropriate random noise. In this way, having chosen to make our primary and sensitivity analysis information anchored, we can derive a corresponding point estimator whose long‐run variance is the information‐anchored variance.

If we wish to do this, we can proceed as follows. Recall that reference‐based methods calculate the means of the missing values for each patient as linear combinations of the estimated treatment means at each time point under randomized arm MAR. Assume *J* follow‐up visits, and denote these estimated means by the 2*J*×1 column vector ***μ***, with estimated covariance matrix $\hat{V}$. It follows that, for some 2*J*×1 column vector **L**, the maximum likelihood reference‐based treatment estimate is given by **L** ^T^ ***μ***, with associated estimated empirical variance ${\hat{\sigma }}_{\mathrm{ML}}^{2}=L^{T}\hat{V}L$. If we denote the information‐anchored variance by ${\hat{\sigma }}_{\mathrm{IA}}^{2},$ take a draw from $N(0,{\hat{\sigma }}_{\mathrm{IA}}^{2}-{\hat{\sigma }}_{\mathrm{ML}}^{2})$ and add this to the treatment estimate that is obtained from the reference‐based analysis by multiple imputation, this will result in an estimate with the information‐anchored variance in a long‐run sense. In practice ${\hat{\sigma }}_{\mathrm{ML}}^{2}$ could also be estimated by using one of the implementations of Lu (2014), Tang (2017) or Liu and Pang (2016). In applications, however, we do not think that this step is typically worthwhile. Note also that with the *δ*‐method ${\hat{\sigma }}_{\mathrm{IA}}^{2}$ is well approximated by ${\hat{\sigma }}_{\mathrm{ML}}^{2},$ so it is not necessary.

This paper has focused on the analysis of a longitudinal measure of a continuous outcome. For generalized linear models, if we perform controlled multiple imputation on the linear predictor scale, then we can apply the theory that was developed here on the linear predictor scale. This suggests that, for generalized linear models, controlled multiple imputation will be approximately information anchored; preliminary simulations support this, and work in this area is continuing. We note, however, that issues may arise with non‐collapsibility when combining the component models in this setting. For survival data, we need to define the reference‐based assumptions. This has been done in Atkinson (2018), which also contains simulation results suggesting promising information anchoring properties for Rubin's rules in this setting.

When conducting class 1 sensitivity analyses via multiple imputation a natural question might be how many imputations to conduct. As remarked in the proof of theorem 1 in the on‐line appendix B.2, the number of imputations does not materially affect the information anchoring performance of Rubin's variance estimate. Thus we recommend determining the number of imputations that are required for primary analysis (under MAR) based on the required precision; these should estimate the information anchored variance with similar precision in sensitivity analysis. To establish the number of imputations that are required to achieve a specific level of precision under MAR Rubin (1987) showed that the relative variance, i.e. the efficiency of an estimate using only *K* imputations compared with an infinite number, is approximately 1+*λ*/*K*, where *λ* is the fraction of missing information. As discussed in Carpenter and Kenward (2008), pages 86–87, 5–10 imputations are sufficient to obtain a reasonably accurate answer for most applications. For more critical inferences, at least 50–100 imputations are recommended (see Carpenter and Kenward (2013), pages 54–55).

Of course, to obtain information‐anchored analyses multiple imputation does not have to be used. In principle we can perform information‐anchored analysis by calculating the variance directly from the information anchoring formula. However, to do this we need to calculate the expected value of the design variance when we actually observe data under the sensitivity assumption. When the approach is used with its full flexibility (with different assumptions for different groups of patients) this is awkward. Multiple imputation provides a much more direct, computationally general, accessible approach for busy trialists, without the need for sophisticated one‐off programming which is often required to fit data missing not at random pattern mixture models or other data missing not at random models directly.

In conclusion, we believe that sensitivity analysis via controlled multiple imputation provides an accessible practical approach to exploring the robustness of inference under the primary assumption to a range of accessible, contextually plausible alternative scenarios. It is increasingly being used in the regulatory world (see, for example, the Drug Information Association pages at www.missingdata.org.uk, and the code therein, Philipsen *et al*. (2015), Jans *et al*. (2015), Billings *et al*. (2018), Atri *et al*. (2018), O’Kelly and Ratitch (2014) and references therein). Our aim has been to provide a more formal underpinning. Information anchoring is a natural principle for such analysis, and we have shown that this is an automatic consequence of using multiple imputation in this setting.
