# Supplementary material for: Information‐anchored sensitivity analysis: theory and application
Source: J R Stat Soc Ser A Stat Soc. 2018 Nov 16;182(2):623–45. doi: 10.1111/rssa.12423 (PMC6378615; doi:10.1111/rssa.12423)
Supplement: Supplementary file 1 [file RSSA-182-623-s001.pdf]

A. Appendix A: Algorithm for reference-based multiple imputation

For a continuous outcome, the generic algorithm of Carpenter *et al.* (2013) can be summarized in full as follows:

(a) Separately for each treatment arm take all the observed data, and assuming MAR, fit a multivariate normal (MVN) distribution with an unstructured mean (i.e. a separate mean for each of the baseline and post-randomisation observation times) and variance covariance matrix using a Bayesian approach with an improper prior for the mean and an uninformative Jeffreys prior for the covariance matrix.

(b) Draw a mean vector and covariance matrix from the posterior distribution for each treatment arm. Specifically we use the Markov-Chain Monte Carlo (MCMC) method to draw from the appropriate Bayesian posterior, with a sufficient burn-in and update the chain sufficiently in-between to ensure subsequent draws are independent. The sampler is initiated using the Expectation-Maximization (EM) algorithm.

(c) Use the draws in step 2 to form the joint distribution of each deviating individual's observed and missing outcome data as required. This can be done under a range of assumptions, in order to explore the robustness of inference about treatment effects. The options presented in Carpenter *et al.* (2013) that each translate to a relevant assumption are described in Table 1.

(d) Construct the conditional distribution of missing (post-deviation) given observed outcome data for each individual who deviated, using their joint distribution formed

in step 3. Sample their missing post-deviation data from this conditional distributions to create a completed data set.

(e) Repeat steps 2–4 K times, resulting in K imputed data sets.

We now describe how step 3 works under 'jump to reference'. This leads to a brief presentation of the approach for the other options. Suppose there are two arms, active (indexed below by a) and reference (indexed below by r). In step 2, denote the current draw from the posterior for the $1+J$ reference arm means and variance-covariance matrix by $\mu_{r,0}, \ldots \mu_{r,J}$, and $\boldsymbol{\Sigma}_r$. Use the subscript a for the corresponding draws from the other arm in question (which will depend on the arm chosen as reference for the analysis at hand).

Under 'jump to reference', suppose patient i is not randomised to the reference arm and their last observation, prior to deviating, is at time d_i, $d_i \in (1, \ldots, J-1)$. The joint distribution of their observed and post-withdrawal outcomes is multivariate normal with mean

$$\tilde{\boldsymbol{\mu}}_i = (\mu_{a,0}, \ldots, \mu_{a,d_i}, \mu_{r,d_i+1}, \ldots, \mu_{r,J})^T;$$

that is post-deviation they 'jump to reference'.

We construct the new covariance matrix for these observations as follows. Denote the covariance matrices from the reference arm (without deviation) and the other arm in question (without deviation), partitioned at time d_i according to the pre- and post-deviation measurements, by:

$$\text{Reference } \boldsymbol{\Sigma}_r = \begin{bmatrix} \mathbf{R}_{11} & \mathbf{R}_{12} \\ \mathbf{R}_{21} & \mathbf{R}_{22} \end{bmatrix} \text{ and other arm: } \boldsymbol{\Sigma}_a = \begin{bmatrix} \mathbf{A}_{11} & \mathbf{A}_{12} \\ \mathbf{A}_{21} & \mathbf{A}_{22} \end{bmatrix}.$$

We want the new covariance matrix, $\boldsymbol{\Sigma}$ say, to match that from the active arm for the pre-deviation measurements, and the reference arm for the *conditional* components for the post-deviation given the pre-deviation measurements. This also guarantees positive definiteness of the new matrix, since $\boldsymbol{\Sigma}_r$ and $\boldsymbol{\Sigma}_a$ are positive definite. That is, we want

$$\boldsymbol{\Sigma} = \begin{bmatrix} \boldsymbol{\Sigma}_{11} & \boldsymbol{\Sigma}_{12} \\ \boldsymbol{\Sigma}_{21} & \boldsymbol{\Sigma}_{22} \end{bmatrix},$$

subject to the constraints

$$\begin{aligned} \boldsymbol{\Sigma}_{11} &= \mathbf{A}_{11}, \\ \boldsymbol{\Sigma}_{21}\boldsymbol{\Sigma}_{11}^{-1} &= \mathbf{R}_{21}\mathbf{R}_{11}^{-1}, \\ \boldsymbol{\Sigma}_{22} - \boldsymbol{\Sigma}_{21}\boldsymbol{\Sigma}_{11}^{-1}\boldsymbol{\Sigma}_{12} &= \mathbf{R}_{22} - \mathbf{R}_{21}\mathbf{R}_{11}^{-1}\mathbf{R}_{12}. \end{aligned}$$

The solution is:

$$\begin{aligned} \boldsymbol{\Sigma}_{11} &= \mathbf{A}_{11}, \\ \boldsymbol{\Sigma}_{21} &= \mathbf{R}_{21}\mathbf{R}_{11}^{-1}\mathbf{A}_{11}, \\ \boldsymbol{\Sigma}_{22} &= \mathbf{R}_{22} - \mathbf{R}_{21}\mathbf{R}_{11}^{-1}(\mathbf{R}_{11} - \mathbf{A}_{11})\mathbf{R}_{11}^{-1}\mathbf{R}_{12}. \end{aligned}$$

Under 'jump to reference' we have now specified the joint distribution for a patient's pre- and post-deviation outcomes, when deviation is at time d_i. This is what we require

for step 4. For 'copy increments in reference' we use the same $\mathbf{\Sigma}$ as for 'jump to reference' but now

$$\begin{aligned}\boldsymbol{\mu}_i &= \{\mu_{a,0}, \ldots, \mu_{a,d_i-1}, \mu_{a,d_i}, \mu_{a,d_i} + (\mu_{r,d_i+1} - \mu_{r,d_i}),\\ &\qquad \mu_{a,d_i} + (\mu_{r,d_i+2} - \mu_{r,d_i}), \ldots\}^T .\end{aligned}$$

For 'last mean carried forward', $\mathbf{\Sigma}$ equals the covariance matrix from the randomisation arm. The important change is the way we put together $\boldsymbol{\mu}$. Thus, for patient i in arm a under 'last mean carried forward',

$$\boldsymbol{\mu}_i = (\mu_{a,0}, \ldots, \mu_{a,d_i-1}, \mu_{a,d_i}, \mu_{a,d_i}, \ldots \ldots)^T; \quad \mathbf{\Sigma} = \mathbf{\Sigma}_a.$$

Finally for 'copy reference' the mean and covariance both come from the reference (typically, but not necessarily, control) arm, irrespective of deviation time. A SAS macro implementing this approach can be downloaded from,
www.missingdata.org.uk (Roger, 2012) and Stata software from
https://ideas.repec.org/c/boc/bocode/s457983.html (Cro, 2015; Cro *et al.*, 2016).

B. Appendix B: Proofs

B.1. Proof of Proposition 1

Here we outline the argument for Proposition 1. Consider the baseline (time 1) and $J-1$ follow-up setting where $Y_{z,i,j}$ denotes the continuous outcome measure for patient i in arm z ($z = a$ indicates active arm allocation and $z = r$ reference arm allocation) at time j for $i = 1, ..., n$ and $j = 1, ..., J$. $n_{d,j}$ patients deviate at time j in a monotone fashion, for $j > 1$ such that $n_d = \sum_{j=2}^{J} n_{d,j}$. Interest lies in the unadjusted mean treatment group difference at time J. Conditioning on $n_{d,j}$ for $j > 1$, the expected value of the treatment estimate at time J when the post-deviation data can be observed is,

$$\left(\frac{n_o}{n}\mu_{a,J} + \sum_{j=2}^{J}\frac{n_{d,j}}{n}\mu_{d,j,J}\right) - \mu_{r,1}.$$

The variance of this estimate is calculated using the usual sample variance formula as,

$$\frac{\frac{1}{n-1}\sum_{i=1}^{n}\left(Y_{r,i,J} - \bar{Y}_{r,J}\right)^2}{n} + \frac{\frac{1}{n-1}\sum_{i=1}^{n}\left(Y_{a,i,J} - \frac{n_o}{n}\bar{Y}_{a,J,o} - \sum_{j=2}^{J}\frac{n_{d,j}}{n}\bar{Y}_{a,J,d,j}\right)^2}{n}$$

where $\bar{Y}_{r,J} = \frac{1}{n}\sum_{i=1}^{n} Y_{r,i,J}$, $\bar{Y}_{a,J,o} = \frac{1}{n_o}\sum_{i\in o} Y_{a,i,J}$ and $\bar{Y}_{a,J,d,j} = \frac{1}{n_{d,j}}\sum_{i\in d,j} Y_{a,i,J}$ for $j = 2, ..., J$. When expanding this expression and letting $(n-1) \to n$ this has expected value,

$$E\left[\hat{V}_{\text{full, sensitivity}}\right] = \frac{\sigma^2_{J,J}}{n} + \frac{\sigma^2_{J,J}}{n} + \sum_{j=2}^{J}\frac{n_o n_{d,j}\Delta^2_{d,j}}{n^3} + \sum_{p=2}^{J}\sum_{\substack{q=2\\ q\neq p}}^{J}\frac{n_{d,p}n_{d,q}\Delta^2_{d,p,q}}{n^3}$$

where $\Delta_{d,j} = \mu_{a,J} - \mu_{d,j,J}$, $\Delta_{d,p,q} = \mu_{d,p,J} - \mu_{d,q,J}$, $\mu_{d,j,J}$ is the mean proposed under the controlled scenario at time J, for patients who deviate at time j and $\mu_{d,p,J}$ and

$\mu_{d,q,J}$ are the means proposed under the controlled scenario at time J, for patients who deviate at times p and q for $p = 2, ..., J$ and $q = 2, ..., J$. For the δ-method of MI where imputed values at final time J are edited by $(J + 1 - j)\delta$, for patients who deviate at time j, we replace $\Delta_{d,j} = \mu_{a,J} - \mu_{d,j,J} = (J + 1 - j)\delta$ and $\Delta_{d,p,q} = \mu_{d,p,J} - \mu_{d,q,J} = (J + 1 - p)\delta - (J + 1 - q)\delta$.

B.2. Proof of Theorem 1

Let $\mathcal{D}$ and $\mathcal{O}$ define the sets of indices for the patients who do and do not deviate in the active arm respectively. Further let $\mathcal{DJ}$ denote the set of indices for deviating patients who deviate at time j, so that the total number of deviating patients in the active arm $n_d = \sum_{j=2}^{J} n_{d,j}$. The follow-up outcome data at the final time point for the reference patients are contained in the vector $\mathbf{Y}_{r,J} = (Y_{r,1,J}, ...Y_{r,n,J})^T$. The final visit outcome data for the observed non-deviating active patients are contained in the vector $\mathbf{Y}_{a,J,o} = \{Y_{a,i,J}; i \in \mathcal{O}\}^T$.

We suppose that each deviating patient has two potential outcomes at time J: the one that would occur if they remain on active treatment post-deviation (primary on-treatment data model, indexed below with the subscript $_P$) and the other that would occur under the controlled sensitivity scenario data model (indexed below with the subscript $_S$). The potentially observable primary on-treatment data for the n_d deviating patients at time J are contained in the vector $\mathbf{Y}_{a,J,P,d}$ and the alternative outcome data under the sensitivity scenario in the vector $\mathbf{Y}_{a,J,S,d}$. Define $\mathbf{Y} = (\mathbf{Y}_{r,J}, \mathbf{Y}_{a,J,o}, \mathbf{Y}_{a,J,P,d}, \mathbf{Y}_{a,J,S,d})^T$ as the collection of observed and potentially observable outcome data at time J, which has dimensions $[(n + n_o + 2n_d) \times 1]$.

For each deviating patient we can only observe one of the potential outcomes, either primary on-treatment or under the sensitivity scenario. Consider two $[(n + n_o + 2n_d) \times (n + n_o + 2n_d)]$ matrices, $\mathbf{D_P}$ and $\mathbf{D_S}$ of 0's and 1's such that $\mathbf{D_P Y}$ gives the $[(n + n_o + 2n_d) \times 1]$ on-treatment (primary) data and $\mathbf{D_S Y}$ gives the $[(n + n_o + 2n_d) \times 1]$ sensitivity scenario data at time J.

Let $\mathbf{a}$ be a $[(n + n_o + 2n_d) \times 1]$ vector such that $\mathbf{a^T D_P Y}$ returns the primary on-treatment treatment estimate and $\mathbf{a^T D_S Y}$ returns the sensitivity scenario treatment estimate. When the deviating patients experience primary on-treatment behaviour post-deviation and are fully observed the expectation of the variance of the primary on-treatment estimand can be expressed as,

$$\mathrm{E}\left[\hat{V}_{\text{full, primary}}\right] = \mathrm{E}\left[\, V\left(\mathbf{a^T D_P Y}\right)\right] = \mathrm{E}\left[\mathbf{a^T D_P}\, V\,(\mathbf{Y})\mathbf{D_P^T a}\right] = \mathbf{a^T D_P \Sigma D_P^T a}. \quad (7)$$

Under the conditions of Proposition 1 and using Corollary 1 and 2, the variance estimator for the sensitivity estimand where post-deviation data are fully observed can be expressed as,

$$E\left[\hat{V}_{\text{full, sensitivity}}\right] = \mathbf{a^T D_P \Sigma D_P^T a} + O(n^{-2}). \quad (8)$$

We now suppose that post-deviation data are unobserved, i.e. the potentially observable primary on-treatment and sensitivity scenario entries in $\mathbf{Y}$ are missing for the n_d active patients. We alternatively multiply impute these outcomes, using primary on-treatment (MAR) imputation and imputation under the sensitivity scenario. This gives

K 'complete' data samples $\mathbf{Y}_k$, of size $[(n + n_o + 2n_d) \times 1]$. For this we need appropriate imputation distributions for each missing data pattern under each scenario, with suitable posteriors for the included parameters.

Under our primary on-treatment assumption (MAR), the imputation model for patients deviating at time j, for each $j > 1$ is formed from the regression of $\mathbf{Y}_{a,J,o}$ on $\mathbf{P}_{a,o,j}$ where $\mathbf{P}_{a,o,j}$ is the design matrix for the imputation model, which contains the values of the $1, ..., j-1$ outcomes and covariates included in the imputation model (excluding treatment) for the n_o observed active patients, along with a vector of 1's to include an intercept in the model. This is appropriate since we are not imputing any interim missing outcomes here. We only consider monotone missing data patterns. We are interested in the treatment effect at time J. As described by Carpenter and Kenward (2013, p. 77–78), under MAR, each of the regressions will be validly estimated from those observed in the data set. The parameter estimates for the primary on-treatment (MAR) imputation model for the $n_{d,j}$ patients missing outcomes j to J for each $j > 1$ are found as $\hat{\boldsymbol{\beta}}_{primary,j} = (\mathbf{P}_{a,o,j}^T \mathbf{P}_{a,o,j})^{-1} \mathbf{P}_{a,o,j}^T \mathbf{Y}_{a,J,o}$ with assumed known covariance matrix $\mathbf{V}_{primary,j} = (\mathbf{P}_{a,o,j}^T \mathbf{P}_{a,o,j})^{-1} \sigma_j^2$.

We assume the large sample posterior for the parameter estimates for the primary on-treatment imputation model, denoted $\hat{\boldsymbol{\beta}}_{primary,j}$, is normal and centered on the ML estimator $\hat{\boldsymbol{\beta}}_{primary,j}$ with covariance matrix $\mathbf{V}_{primary,j}$. That is,

$$\hat{\boldsymbol{\beta}}_{primary,j} | Y_{a,J,o} \sim N(\hat{\boldsymbol{\beta}}_{primary,j}; \mathbf{V}_{primary,j}).$$

The primary on-treatment imputation model for active patient i deviating at time j, for each $j > 1$ and imputation k can therefore be expressed as,

$$\tilde{Y}_{a,i,J,k} | \mathbf{Y}_{a,J,o} = \mathbf{P}_{a,d,j,i} \left[\hat{\boldsymbol{\beta}}_{primary,j} + \mathbf{b}_{primary,j,k} \right] + e_{i,j,k} \text{ for } i \in \{\mathcal{DJ}\},$$

where, $\mathbf{b}_{primary,j,k} \sim N(0, \mathbf{V}_{a,o,j}), e_{i,j,k} \sim N(0, \sigma_j^2)$ and $\mathbf{P}_{a,d,j,i}$ contains the values of the $1, ..., j-1$ outcomes and covariates included in the imputation model (excluding treatment, plus a 1 for the intercept) for each deviating active patient i, who deviates at time j.

For sensitivity analysis we conduct imputation under the proposed sensitivity scenario and assume the large sample posterior for the imputation parameters for the $n_{d,j}$ patients missing outcomes j to J for each $j > 1$, $\hat{\boldsymbol{\beta}}_{sensitivity,j}$ is normal and centered on the ML estimator $\hat{\boldsymbol{\beta}}_{sensitivity,j}$ with known covariance matrix $\mathbf{V}_{sensitivity,j}$, that is for each $j > 1$,

$$\hat{\boldsymbol{\beta}}_{sensitivity,j} | \mathbf{Y}_{sensitivity,J} \sim N(\hat{\boldsymbol{\beta}}_{sensitivity,j}; \mathbf{V}_{sensitivity,j}),$$

where $\mathbf{Y}_{sensitivity,J}$ consists of the relevant observed outcome data under the particular sensitivity scenario setting of interest. The imputation model used in the sensitivity analysis for active patient i deviating following time j, for each $j > 1$ and imputation k can therefore be expressed as,

$$\breve{Y}_{a,i,J,k} | \mathbf{Y}_{sensitivity,J} = \mathbf{P}_{a,d,j,i} \left[\hat{\boldsymbol{\beta}}_{sensitivity,j} + \mathbf{b}_{sensitivity,j,k} \right] + e_{i,j,k} \text{ for } i \in \{\mathcal{DJ}\},$$

where, $\mathbf{b}_{sensitivity,j,k} \sim N(0, \mathbf{V}_{sensitivity,j})$ and $e_{i,j,k} \sim N(0, \sigma_j^2)$. Under the assumption of equal variance-covariance matrix of baseline and follow-up by treatment arm we consequently assume the same variance for the residuals in the primary and sensitivity imputation models for patients deviating at the same time j, for each $j > 1$.

We are interested in imputation inference for, $\frac{1}{K}\sum_{k=1}^{K} \mathbf{a^T D_P Y}_k$ or $\frac{1}{K}\sum_{k=1}^{K} \mathbf{a^T D_S Y}_k$. Letting the number of imputations, $K \to \infty$, the variance of our MI treatment estimate as estimated by Rubin's rules is, $\hat{V}_{\text{MI, primary}} = \hat{W}_{\text{primary}} + \hat{B}_{\text{primary}}$ or $\hat{V}_{\text{MI, sensitivity}} = \hat{W}_{\text{sensitivity}} + \hat{B}_{\text{sensitivity}}$ where under the conditions required in the proposition,
$E\left[\hat{W}_{\text{primary}}\right] = E\left[\frac{1}{K}\sum_{k=1}^{K} \mathbf{a^T D_P \hat{\Sigma}_k D_P^T a}\right] \to \mathbf{a^T D_P \Sigma D_P^T a}$ and,
$E\left[\hat{W}_{\text{sensitivity}}\right] = E\left[\frac{1}{K}\sum_{k=1}^{K} \mathbf{a^T D_S \hat{\Sigma}_k D_S^T a}\right] \to \mathbf{a^T D_P \Sigma D_P^T a} + O(n^{-2})$.

Under primary (on-treatment) imputation,

$$\hat{B}_{\text{primary}} = \frac{1}{K-1}\sum_{k=1}^{K}\left[\sum_{j=2}^{J} \pi_{d,j}\left(\bar{e}_{j,k} - \bar{e}_j\right) + \pi_{d,j}\left(\bar{\mathbf{P}}_{a,d,j}\mathbf{b}_{primary,j,k} - \bar{\mathbf{P}}_{a,d,j}\bar{\mathbf{b}}_{primary,j}\right)\right]^2$$

where $\bar{e}_{j,k} = \frac{1}{n_{d,j}}\sum_{i\in\mathcal{DJ}} e_{i,j,k}$, $\bar{e}_j = \frac{1}{K}\sum_{k=1}^{K}\bar{e}_{j,k}$, $\bar{\mathbf{P}}_{a,d,j} = \frac{1}{n_{d,j}}\sum_{i\in\mathcal{DJ}}\mathbf{P}_{a,d,j,i}$ and $\bar{\mathbf{b}}_{primary,j} = \frac{1}{K}\sum_{k=1}^{K}\mathbf{b}_{primary,j,k}$. Which has expectation,

$$E\left[\hat{B}_{\text{primary}}\right] = \sum_{j=2}^{J}\pi_{d,j}^2\left[\frac{\sigma_j^2 + n_{d,j}\bar{\mathbf{P}}_{a,d,j}\mathbf{V}_{primary,j}\bar{\mathbf{P}}_{a,d,j}^T}{n_{d,j}}\right].$$

When imputation is conducted under the sensitivity scenario,

$$\hat{B}_{\text{sensitivity}} = \frac{1}{K-1}\sum_{k=1}^{K}\left[\sum_{j=2}^{J} \pi_{d,j}\left(\bar{e}_{j,k} - \bar{e}_j\right) + \pi_{d,j}\left(\bar{\mathbf{P}}_{a,d,j}\mathbf{b}_{sensitivity,j,k} - \bar{\mathbf{P}}_{a,d,j}\bar{\mathbf{b}}_{sensitivity,j}\right)\right]^2,$$

where $\bar{\mathbf{b}}_{sensitivity,j} = \frac{1}{K}\sum_{k=1}^{K}\mathbf{b}_{sensitivity,j,k}$. Which has expectation,

$$E\left[\hat{B}_{\text{sensitivity}}\right] = \sum_{j=2}^{J}\pi_{d,j}^2\left[\frac{\sigma_j^2 + n_{d,j}\bar{\mathbf{P}}_{a,d,j}\mathbf{V}_{sensitivity,j}\bar{\mathbf{P}}_{a,d,j}^T}{n_{d,j}}\right].$$

The information-anchored variance can be expressed as,

$$E\left[\hat{V}_{\text{anchored}}\right] = \frac{E\left[\hat{V}_{\text{full, sensitivity}}\right]\left(E\left[\hat{W}_{\text{primary}}\right] + E\left[\hat{B}_{\text{primary}}\right]\right)}{E\left[\hat{V}_{\text{full, primary}}\right]} = E\left[\hat{V}_{\text{full, sensitivity}}\right]\left[1 + \frac{E\left[\hat{B}_{\text{primary}}\right]}{E\left[\hat{W}_{\text{primary}}\right]}\right].$$

Since $E\left[\hat{W}_{\text{primary}}\right] = E\left[\hat{V}_{\text{full, primary}}\right]$ and using (7) and (8) that is,

$$\begin{aligned} E\left[\hat{V}_{\text{anchored}}\right] &= \mathbf{a^T D_P \Sigma D_P^T a} + O(n^{-2}) + \frac{E\left[\hat{B}_{\text{primary}}\right]}{E\left[\hat{W}_{\text{primary}}\right]}\left[\mathbf{a^T D_P \Sigma D_P^T a} + O(n^{-2})\right] \\ &= \mathbf{a^T D_P \Sigma D_P^T a} + O(n^{-2}) + E\left[\hat{B}_{\text{primary}}\right] + \frac{E\left[\hat{B}_{\text{primary}}\right]}{E\left[\hat{W}_{\text{primary}}\right]} O(n^{-2}). \end{aligned}$$

If Rubin's rules are information-anchoring and preserve the information loss in the primary analysis under MAR then the following holds,

$$E\left[\hat{W}_{\text{sensitivity}}\right]+E\left[\hat{B}_{\text{sensitivity}}\right]\approx \mathbf{a^T D_P \Sigma D_P^T a}+O(n^{-2})+E\left[\hat{B}_{\text{primary}}\right]+\frac{E\left[\hat{B}_{\text{primary}}\right]}{E\left[\hat{W}_{\text{primary}}\right]}O(n^{-2}).$$

That is,

$$\begin{aligned}\mathbf{a^T D_P \Sigma D_P^T a}+O(n^{-2})+E\left[\hat{B}_{\text{sensitivity}}\right]\approx \quad & \mathbf{a^T D_P \Sigma D_P^T a}+O(n^{-2}) \\ & +E\left[\hat{B}_{\text{primary}}\right]+\frac{E\left[\hat{B}_{\text{primary}}\right]}{E\left[\hat{W}_{\text{primary}}\right]}O(n^{-2}).\end{aligned}$$

After simplification and rearrangement this becomes,

$$0\approx E\left[\hat{B}_{\text{primary}}\right]-E\left[\hat{B}_{\text{sensitivity}}\right]+\frac{E\left[\hat{B}_{\text{primary}}\right]}{E\left[\hat{W}_{\text{primary}}\right]}\left[O(n^{-2})\right].$$

Which is,

$$0\approx\sum_{j=2}^{J}\left[\pi_{d,j}^2\bar{\mathbf{P}}_{a,d,j}\left(\mathbf{V}_{primary,j}-\mathbf{V}_{sensitivity,j}\right)\bar{\mathbf{P}}_{a,d,j}^T\right]+\frac{E\left[\hat{B}_{\text{primary}}\right]}{E\left[\hat{W}_{\text{primary}}\right]}\left[O(n^{-2})\right]$$

This gives the required result in the longitudinal trial setting with monotone missingness in the active treatment arm with $K=\infty$. In practice $K\neq\infty$, however the information-anchoring approximation results will still hold for finite K. For finite K the variance of our MI treatment estimate as estimated by Rubin's rules is, $\hat{V}_{\text{MI, primary}}=\hat{W}_{\text{primary}}+\left(1+\frac{1}{K}\right)\hat{B}_{\text{primary}}$ or $\hat{V}_{\text{MI, sensitivity}}=\hat{W}_{\text{sensitivity}}+\left(1+\frac{1}{K}\right)\hat{B}_{\text{sensitivity}}$. We will therefore have additional terms in the difference between Rubin's variance estimator and the ideal information-anchored variance, but these will also be very small. They will be the same order of the terms already presented multiplied by K^{-1}, hence indeed smaller. Thus following the reasons discussed in the main text the approximation remains with finite K.

We note that when we relax the equal variance by trial arm assumption, we can no longer assume the variance of the residuals in the primary de jure imputation model for patients with missingness pattern j matches the variance of the residuals in the sensitivity de facto imputation model for patients with missingness pattern j, for each missing data pattern j.

In this setting we denote the variance of the residuals in the primary on-treatment imputation model for patients missing outcomes $j,...,J$ as $\sigma_{P,j}^2$ and in the sensitivity imputation model as $\sigma_{S,j}^2$ for $j>1$. Then the information-anchoring performance of Rubin's MI variance estimator is driven by,

$$0\approx\sum_{j=2}^{J}\pi_{d,j}^2\left[\frac{\sigma_{P,j}^2-\sigma_{S,j}^2}{n_{d,j}}+\bar{\mathbf{P}}_{a,d,j}\left(\mathbf{V}_{primary,j}-\mathbf{V}_{sensitivity,j}\right)\bar{\mathbf{P}}_{a,d,j}^T\right]+\frac{E\left[\hat{B}_{\text{primary}}\right]}{E\left[\hat{W}_{\text{primary}}\right]}\left[O(n^{-2})\right].$$

The additional components in the difference between Rubin's variance and the ideal information-anchored variance are driven by the degree of difference in the variance structure of the data by trial arm for each missingness pattern. Since the variance structure is not likely to differ too markedly by trial arm for each missingness pattern, and these extra components are each multiplied by $\pi_{d,j}^2/n_{d,j}$, the overall impact will in practice be relatively small.
